# EZH2-Mediated H3K27me3 Is Involved in Epigenetic Repression of Deleted in Liver Cancer 1 in Human Cancers

**DOI:** 10.1371/journal.pone.0068226

**Published:** 2013-06-27

**Authors:** Sandy Leung-Kuen Au, Carmen Chak-Lui Wong, Joyce Man-Fong Lee, Chun-Ming Wong, Irene Oi-Lin Ng

**Affiliations:** State Key Laboratory for Liver Research and Department of Pathology, Li Ka Shing Faculty of Medicine, the University of Hong Kong, Hong Kong, People’s Republic of China; University of North Carolina School of Medicine, United States of America

## Abstract

Enhancer of zeste homolog 2 (EZH2), the histone methyltransferase of the Polycomb Repressive complex 2 catalyzing histone H3 lysine 27 tri-methylation (H3K27me3), is frequently up-regulated in human cancers. In this study, we identified the tumor suppressor Deleted in liver cancer 1 (DLC1) as a target of repression by EZH2-mediated H3K27me3. DLC1 is a GTPase-activating protein for Rho family proteins. Inactivation of DLC1 results in hyper-activated Rho/ROCK signaling and is implicated in actin cytoskeleton reorganization to promote cancer metastasis. By chromatin immunoprecipitation assay, we demonstrated that H3K27me3 was significantly enriched at the DLC1 promoter region of a DLC1-nonexpressing HCC cell line, MHCC97L. Depletion of EZH2 in MHCC97L by shRNA reduced H3K27me3 level at DLC1 promoter and induced DLC1 gene re-expression. Conversely, transient overexpression of GFP-EZH2 in DLC1-expressing Huh7 cells reduced DLC1 mRNA level with a concomitant enrichment of EZH2 on DLC1 promoter. An inverse relation between EZH2 and DLC1 expression was observed in the liver, lung, breast, prostate, and ovarian cancer tissues. Treating cancer cells with the EZH2 small molecular inhibitor, 3-Deazaneplanocin A (DZNep), restored DLC1 expression in different cancer cell lines, indicating that EZH2-mediated H3K27me3 epigenetic regulation of DLC1 was a common mechanism in human cancers. Importantly, we found that DZNep treatment inhibited HCC cell migration through disrupting actin cytoskeleton network, suggesting the therapeutic potential of DZNep in targeting cancer metastasis. Taken together, our study has shed mechanistic insight into EZH2-H3K27me3 epigenetic repression of DLC1 and advocated the significant pro-metastatic role of EZH2 via repressing tumor and metastasis suppressors.

## Introduction

Deregulation of upstream epigenetic regulatory proteins promotes epigenetic alterations and contributed to aberrant silencing of tumor suppressor genes in human cancers [1]. Enhancer of zeste homolog 2 (EZH2), the catalytic subunit of Polycomb Repressive Complex 2 (PRC2), is one of the most commonly up-regulated epigenetic regulators in different human cancers [2–5]. EZH2 is a histone methyltransferase that specifically catalyzes histone H3 lysine 27 tri-methylation (H3K27me3), which in turn acts as a repressive histone modification to epigenetically control gene transcription [6,7]. Up-regulation of EZH2 plays a crucial role in malignant progression and was implicated in cancer metastasis [2]. EZH2 functions as an oncogene in different human cancers mainly through epigenetic silencing of tumor and metastasis suppressor genes, including E-cadherin [8], RUNX3 [9], SLIT2 [10], DAB2IP [11], and KLF2 [12]. Recently, we have also reported that EZH2 epigenetically inactivates expressions of multiple tumor and metastasis suppressor microRNAs (miRNAs), such as miR-125b and miR-139 in human hepatocellular carcinoma (HCC), thereby promotes HCC tumorigenicity and metastasis [13]. Identifying novel targets that are silenced by EZH2 will better reveal the molecular roles of EZH2 in cancer metastasis which will be beneficial to the development of chemotherapies targeting EZH2.

DLC1 was identified as a *bona fide* tumor suppressor gene on a recurrently deleted chromosomal region at chromosome 8p21 in HCC [14]. DLC1 is a Rho GTPase-activating protein (RhoGAP) localized at the focal adhesions [15,16], and is specific for controlling the activity of RhoA, B, C and CDC42 [17,18]. The RhoGAP activity of DLC1 negatively regulates these Rho proteins by stimulating their intrinsic GTP hydrolytic activity, thus converts them from the active GTP-bound state to the inactive GDP-bound state. The Rho signaling cascade allows proper control of many biological processes such as cell proliferation [19] and cell movement [20] in normal cells. During malignancy development, the DLC1/Rho pathway is of particular importance owing to its regulation on the actin cytoskeleton related to cancer metastasis. We have shown that loss of DLC1 in HCC activated RhoA, which subsequently activated its downstream effector Rho kinase (ROCK) to remodel the actin cytoskeleton network for cell migration and invasion [21,22]. Other diverse tumor suppressive roles of DLC1 include mediation of caspase-3-dependent apoptosis in HCC model [23], inhibition of VEGF-dependent angiogenesis in prostate cancer model [24], and suppression of clonogenicity in several types of cancers [25]. Down-regulation of DLC1 is commonly shown in a wide range of human malignancies [26] and its loss of expression is classically associated with chromosomal deletion or promoter DNA hypermethylation (Yuan et al 1998; Ng et al 2000; Wong et al 2003; Kim et al 2003). In this present study, we provided the first evidence that DLC1 is a novel target of repression by EZH2-mediated H3K27me3. We further showed inactivation of EZH2 by 3-Deazaneplanocin A (DZNep) that re-expressed DLC1 and remarkably abolished cytoskeletal reorganization and inhibited cell migration in cancer cells. Collectively, our findings suggest that epigenetic silencing of DLC1 is involved in the pro-metastatic function of EZH2 in human cancers.

## Materials and Methods

### Cell lines

SMMC-7721 cell line (Shanghai Institute of Cell Biology) and Huh 7 cell line (Japanese Cancer Research Bank) were maintained in Dulbecco’s modified Eagle’s medium (DMEM)-high glucose. MHCC97L cell line (gift from Prof. Z.Y. Tang of Fudan University, Shanghai) [27] and MIHA cell line (Shanghai Institute of Cell Biology) were maintained in DMEM-high glucose supplemented with sodium pyruvate. HeLa cell line (American Type Culture Collection) was maintained in DMEM-low glucose. CNE2 (American Type Culture Collection) and HCT116 (gift from Prof. B. Vogelstein, Johns Hopkins University School of Medicine, Baltimore, MD) [28] cell lines were maintained in Roswell Park Memorial Institute 1640 medium. All medium was supplemented with 10% fetal bovine serum and 100 units/ml penicillin and streptomycin.

### Bisulfite modification and methylation analysis

Bisulfite modification of genomic DNA extracted from cell lines was performed using EZ DNA Methylation-Direct Kit (ZYMO Research, Orange, CA, USA) according to manufacturer’s instruction. Primers used for amplification of DLC1 promoter after bisulfite modification are: 5’-GTTTTTAGTTAGGATATGGT-3’ (forward) and 5’-ACTTCTTTCTACACATCAAACAC-3’ (reverse) for BS-1 region ; and 5’-TAGAGTTATTAAGAAAAAGAAGGGA-3’ (forward) and 5’-AAAACTAAAATATTTCCCCCAC-3’ (reverse) for BS-2 region. PCR product was cloned into TOPO TA Cloning vector (Invitrogen, Carlsbad, CA) and sequencing of individual clones was performed by Sanger sequencing.

### Chromatin immunoprecipitation (ChIP) assay

ChIP assay was performed using EZ ChIP kit (Millipore, Billerica, MA, USA) as previously described [13]. ChIP-grade antibody against H3K9me3 (ab8898; Abcam, Cambridge, MA, USA), H3K27me3 (07-449; Millipore, Billerica, MA, USA) and EZH2 (#3147; Cell Signaling Technology, Danvers, MA, USA) were used in the assay. Rabbit IgG (Millipore, Billerica, MA, USA) or mouse IgG (Millipore, Billerica, MA, USA) was used as negative control in the assay. Primers spanning -394nt to -325nt upstream of DLC1 transcription start site were used: 5’-CACCTC CGCCAAGTAAATGC-3’ (forward) and 5’-CCGAAAAGTCGCCAACTATTG-3’ (reverse). Quantification of antibody-bound region was done by qPCR performed with ABI Prism 7700 (Applied Biosystems, Carlsbad, CA, USA).

### *In vitro* epigenetic drugs treatment

DZNep (Cayman Chemical, Ann Arbor, MI, USA) was dissolved in dimethyl sulfoxide (DMSO) (Sigma Aldrich, St Louis, MO, USA). 5-Aza-dC (Sigma Aldrich, St Louis, MO, USA) was dissolved in 50% acetic acid. TSA (Sigma Aldrich, St Louis, MO, USA) was dissolved in ethanol. The solvent of the chemicals was used as mock in the corresponding treatment. For DZNep treatment, DZNep (1µM or 10µM) was added to the culture medium for 48 or 72 hours. For 5-Aza-dC treatment, 5-Aza-dC (10 µM) was replenished daily for 72 hours. For TSA treatment, TSA (0.25 µg/mL) was only added to the cells in the last 24 hours of the experiment.

### Cell migration assay

Prior to cell migration assay, 2x10^5^ MHCC97L cells were treated with 1µM DZNep for 48 hours. Mock and DZNep-treated cells (1x10^5^) cells were seeded in the upper chamber of 8µm-pore size Transwell insert (Millipore, Billerica, MA, USA). The lower chamber contained conditioned medium collected from untreated MHCC97L cells to attract cell migration for 18 hours. Migrated cells were fixed with methanol and stained with crystal violet. Three independent views of the Transwell membrane were photographed and number of migrated cells was counted.

### Immunofluorescence (IF) microscopy and Scanning electron microscopy (SEM)

Prior to IF imaging, 2x10^5^ MHCC97L cells were treated with 1µM DZNep for 48 hours. After the treatment, mock and DZNep-treated cells (1x10^5^) cells were trypsinized and seeded on coverslips in DZNep-free culture medium for 24 hours. Cells were fixed with 4% paraformaldehyde in PBS and permeabilized with 0.2% Triton-X-100. Focal adhesions were stained with anti-paxillin antibody (05-417; Millipore, Billerica, MA, USA). F-actin was visualized by staining with FITC-conjugated phalloidin (Sigma Aldrich, St Louis, MO, USA). Nuclei were counterstained with DAPI (Calbiochem, San Diego, CA, USA). IF images were viewed and captured using a Leica Q550CW fluorescence microscope (Leica, Wetzler, Germany). For SEM, mock and DZNep-treated cells were fixed with 1% osmium tetroxide and 2.5% glutaldehyde, followed by stepwise ethanol dehydration. After the step of critical point dry, slides were mounted on silver paste. Images were scanned and captured under ×2,000 magnification using a Hitachi S-4800 FEG Scanning Electron Microscope.

### Statistical analysis

Non-parametric data and continuous parametric data were analyzed by Mann Whiteny U test and *t* test, respectively using the GraphPad Prism version 5.00 (GraphPad Software, San Diego California USA). Tests were considered significant when the *P* value was less than 0.05.

## Results

### H3K27me3 level is differentially enriched on DLC1 promoter in immortalized normal hepatocyte and HCC cell lines

**Figure 1:**
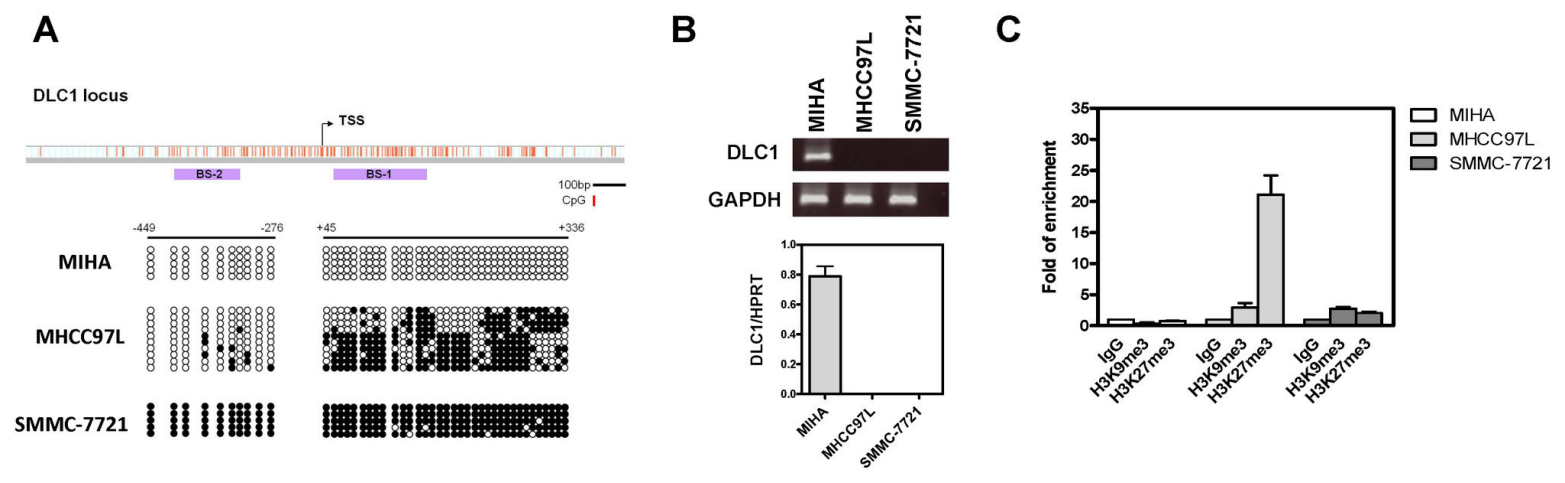
**Promoter DNA methylation** and **histone modification of DLC1 in HCC cell lines.** (**A**) Schematic diagram showing CpG island located at DLC1 locus. DNA methylation status of 45 CpG dinucleotides (BS-1 region included 35 CpG dinulceotides and BS-2 region included 10 CpG dinucleotides) were subject to bisulfite sequencing analysis. MIHA showed complete unmethylation, MHCC97L showed partial methylation and SMMC-7721 showed complete methylation. Open circle represents unmethylated CpG dinucleotides and closed circle represents methylated CpG dinucleotides. Each column represents a single clone being sequenced. (**B**) RT-PCR (upper panel) and qRT-PCR (bottom panel) showing detectable DLC1 mRNA expression in MIHA, but not in MHCC97L and SMMC-7721 cells. (**C**) Chromatin immunoprecipitation (ChIP) assay coupled with qPCR (qChIP) analysis revealed the relative enrichment of H3K9me3 and H3K27me3 on DLC1 promoter region in MIHA, MHCC97L and SMMC-7721 cells. Fold of enrichment of ChIP assay was calculated with reference to IgG control after normalized with the input DNA. Data are represented as mean ± SEM from three independent experiments.

To understand the role of epigenetic deregulation and DLC1 inactivation in HCC, we began with analyzing DLC1 promoter methylation status by bisulfide sequencing in the immortalized normal hepatocyte cell line MIHA and two HCC cell lines SMMC-7721 and MHCC97L. DLC1 promoter was completely unmethylated in MIHA, heterogeneously and partially methylated in MHCC97L, and completely methylated in SMMC-7721 (Figure 1A). The methylation status of DLC1 in MIHA and SMMC-7721 correlated well with their DLC1 transcriptional level. But in MHCC97L, of which the DLC1 promoter was only partially methylated, the complete silencing of DLC1 suggested that other epigenetic regulations may be involved (Figure 1B). We therefore hypothesized that aberrant histone methylation might contribute to DLC1 silencing in MHCC97L. Chromatin immunoprecipitation (ChIP) assay was performed to assess the enrichment of transcriptional repressive histone modifications, H3K9me3 and H3K27me3, on the DLC1 promoter (Figure 1C). We showed that H3K9me3 and H3K27me3 were not detectable in MIHA and this was consistent with the transcriptionally active status of the DLC1 gene in this cell line. In contrast, enrichment of both transcriptional repressive H3K27me3 and H3K9me3 was detected at the DLC1 promoter of SMMC-7721 and MHCC97L cells. Interestingly, H3K27me3 was more abundantly enriched in MHCC97L, as compared with SMMC-7721. This observation indicates that, in addition to the partial DNA methylation, H3K27me3 also took part in the epigenetic silencing to completely transcriptionally inactivate the DLC1 gene in MHCC97L.

### Suppression of EZH2 induced DLC1 reexpression in different human cancer cell lines

**Figure 2:**
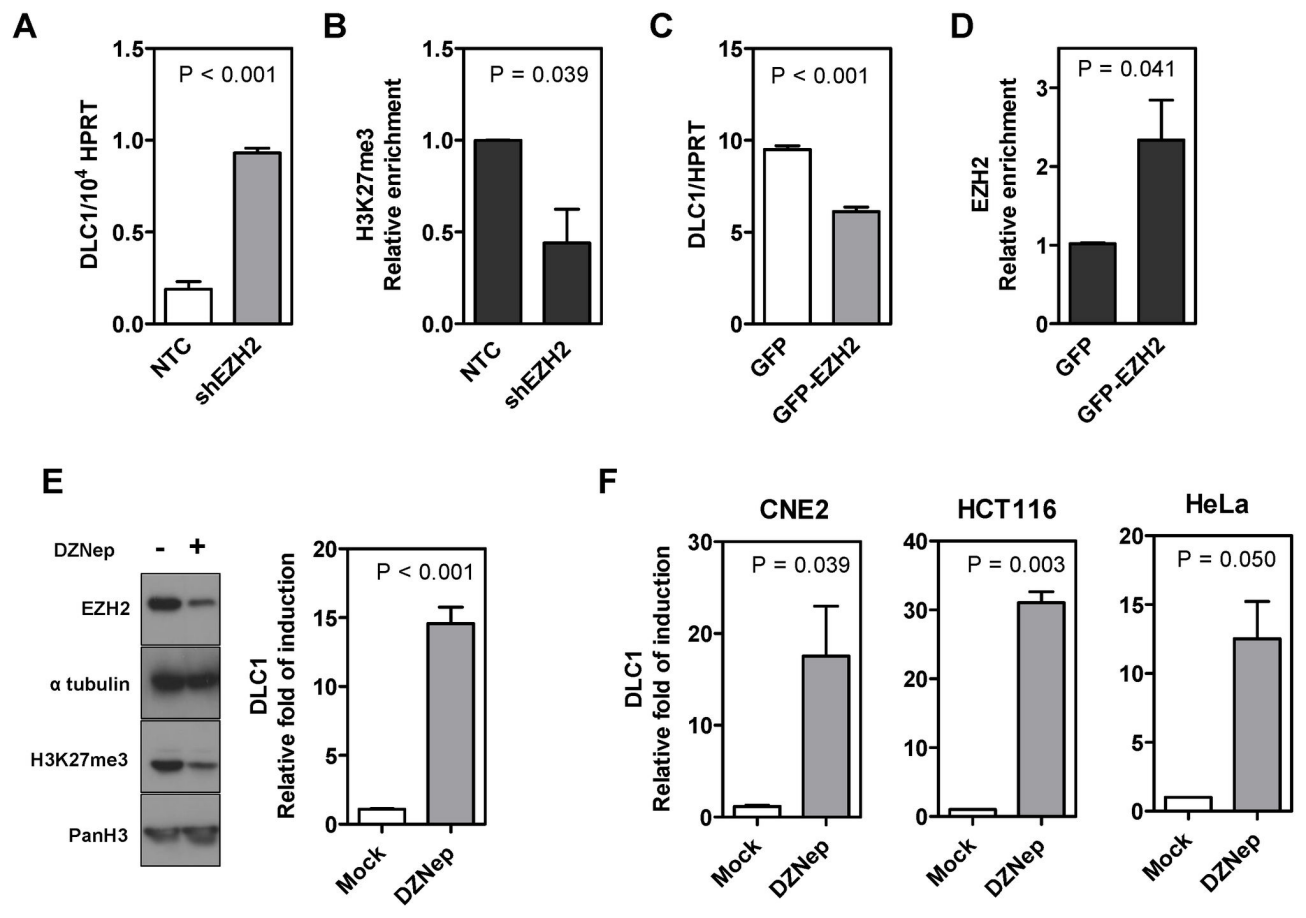
**EZH2-mediated H3K27me3 was involved in epigenetic repression of DLC1 in HCC** and **multiple other human cancers.** (**A**) DLC1 was transcriptionally induced upon stable knockdown of EZH2 in MHCC97L cells. (**B**) qChIP analysis confirmed the depletion of H3K27me3 enrichment on DLC1 promoter upon EZH2 knockdown in MHCC97L cells. Data are represented as mean ± SEM from three independent experiments. (**C**) DLC1 was transcriptionally repressed in Huh7 cells after transient overexpression of GFP-EZH2. (**D**) qChIP analysis revealed a concomitant enrichment of EZH2 at DLC1’s promoter locus upon GFP-EZH2 overexpression in Huh7 cells. Data are represented as mean ± SEM from three independent experiments. (**E**) EZH2 and H3K27me3 expression was reduced upon 1µM DZNep treatment for 48 hours in MHCC97L cells (left panel). DMSO was used as mock treatment. Pan H3 and α-tubulin were loading control of the immunoblot. DZNep treatment transcriptionally induced DLC1 expression in MHCC97L as indicated by qPCR analysis (right panel). (**F**) Different human cancer cells, including the nasopharyngeal carcinoma cell line CNE2, the colorectal carcinoma cell line HCT116 and the cervical adenocarcinoma cell line HeLa were examined for DLC1 re-expression upon DZNep treatment. Treatment of cells with 10µM DZNep reactivated DLC1 expression. *P*-values obtained from *t*-test.

To provide more evidence that EZH2-mediated H3K27me3 directly regulates DLC1, we investigated whether DLC1 expression could be restored upon the knockdown of EZH2. HCC cell lines stably expressing non-target control (NTC) or EZH2 targeting shRNA (shEZH2) were established in our previous study [13]. Upon stable knockdown of EZH2, DLC1 was transcriptionally induced in MHCC97L (Figure 2A). Loss of H3K27me3 on the DLC1 promoter was observed in MHCC97L shEZH2 cells (Figure 2B), indicating that EZH2-mediated H3K27me3 contributes to the suppression of DLC1 in MHCC97L. In DLC1-expressing Huh7 cells, transient overexpression of GFP-EZH2 transcriptionally repressed DLC1 and a concomitant enrichment of EZH2 was detected on the DLC1 promoter (Figure 2C and D Supplementary Figure S1). In line with the EZH2 knockdown model, we further showed that treatment of 3-Deazaneplanocin A (DZNep), a small molecule EZH2 inhibitor [29,30], significantly reduced the EZH2 protein level and H3K27me3 level and induced DLC1 re-expression in MHCC97L cells (Figure 2E). More importantly, we found that EZH2-mediated DLC1 epigenetic silencing was not restricted to HCC. Re-expression of DLC1 upon DZNep treatment was also observed in different cancer cell lines including the nasopharyngeal carcinoma cell line CNE2, colorectal carcinoma cell line HCT116 and cervical adenocarcinoma cell line HeLa (Figure 2F). The above findings indicated that epigenetic silencing of DLC1 by EZH2-mediated H3K27me3 is a common mechanism shared by different human cancers.

### EZH2 and DLC1 expression levels are inversely correlated in human cancer tissues

**Figure 3:**
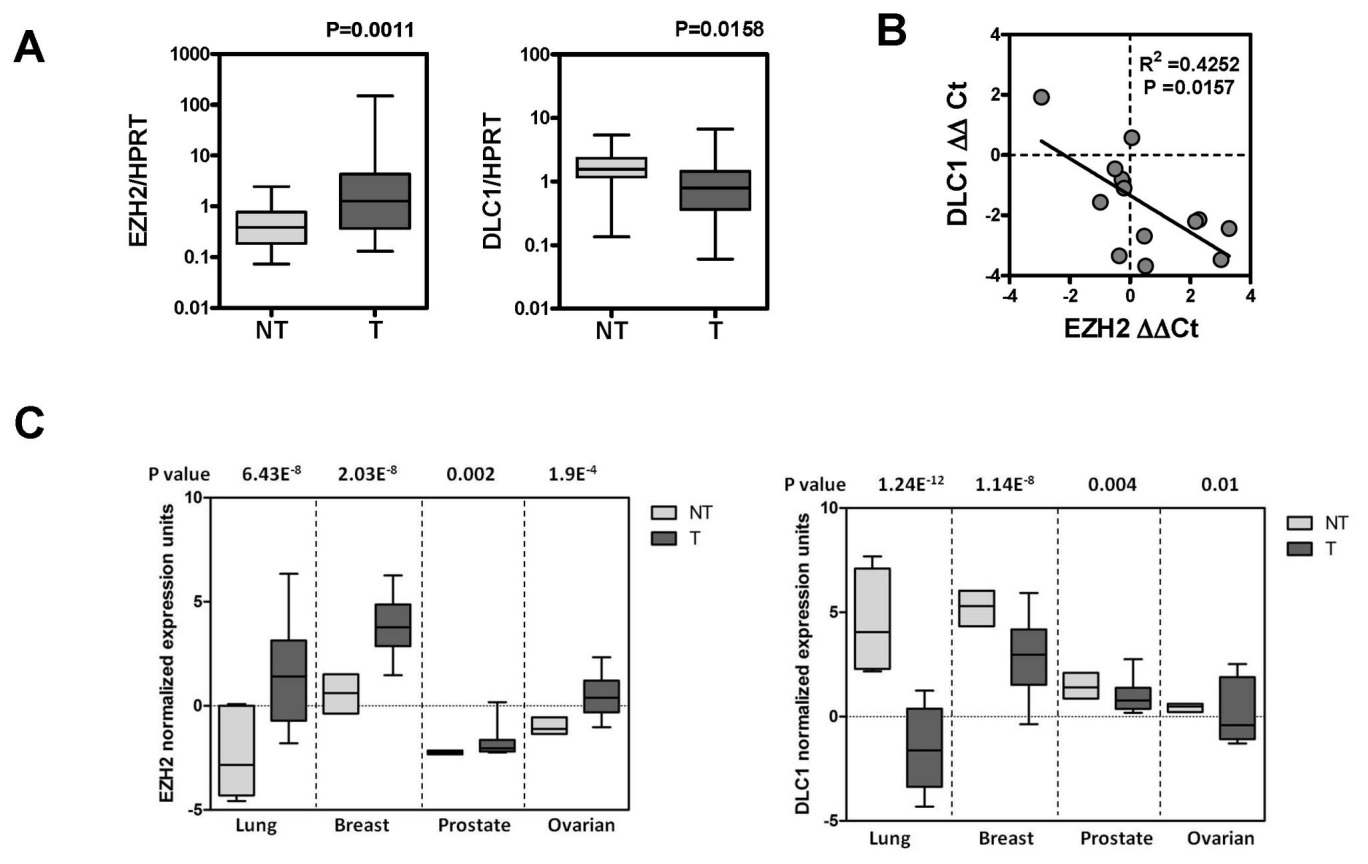
**Inverse correlation between EZH2** and **DLC1 expressions in human cancers.** (**A**) Twenty five-paired HCC samples were examined for EZH2 and DLC1 mRNA expression by qPCR. EZH2 was significantly up-regulated in HCC tumorous (T) tissues than non-tumorous (NT) tissues (left panel). In the same sample cohort, DLC1 was significantly down-regulated in HCC as compared to NT tissues (right panel). *P* values from Wilcoxon matched pair test. (**B**) An inverse correlation was observed between EZH2 and DLC1 expression in a subset of HCC samples without DLC1 promoter methylation. Expression level of EZH2 and DLC1 in paired-HCC samples was represented by ΔΔCt (T_(HPRT Ct –Gene of interest Ct)_-NT_(HPRT Ct – Gene of interest Ct)_). Linear regression analysis was performed using GraphPad Prism5 (La Jolla, CA, USA). (**C**) EZH2 upregulation (left panel) and concomitant DLC1 downregulation (right panel) was consistently observed in the tumorous tissues of different cancers, including lung [32], breast [33], prostate [34] and ovarian [35] cancers. Expression data and *P* values of DLC1 and EZH2 in multiple cancer types was obtained from Oncomine microarray database. Floating bars were shown to illustrate the minimum, median and maximum normalized expression units in non-tumorous (NT) and tumorous (T) tissues.

To provide clinical relevance of our *in vitro* observations, we examined DLC1 mRNA expression in 25-paired primary HCC samples and correlated the expression level with our previous EZH2 mRNA expression data [31]. In this cohort of samples, EZH2 was significantly upregulated while DLC1 was significantly downregulated in the tumorous tissues than the non-tumorous tissues (Figure 3A). Interestingly, a significant inverse correlation between EZH2 and DLC1 was observed in a subset of HCC samples, in which DLC1 promoter was not silenced by DNA methylation [17] (Figure 3B). We also extended our analysis to other human cancers by retrieving the microarray gene expression data of lung [32], breast [33], prostate [34], and ovarian [35] cancers from Oncomine (www.oncomine.org) (Figure 3C). Consistent with the findings from human HCC, concomitant DLC1 down-regulation and EZH2 up-regulation was observed in these malignancies, suggesting the implications of EZH2 up-regulation in silencing DLC1 expression in different human cancers.

### DLC1 is synergistically silenced by H3K27me3 only when its promoter DNA is partially methylated

**Figure 4:**
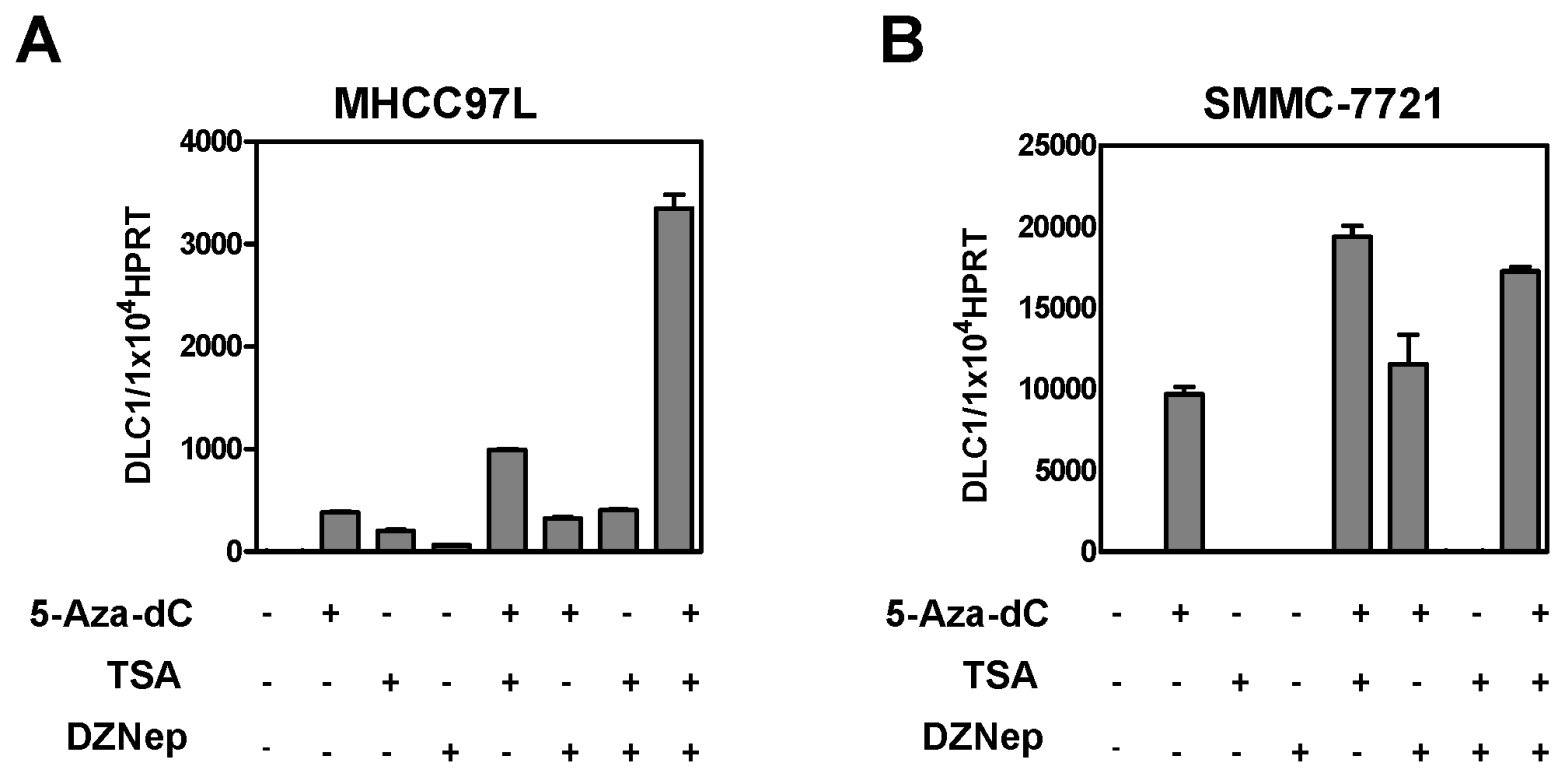
**DLC1 expression was synergistically restored upon DZNep, 5-Aza-dC** and **TSA treatment in MHCC97L but not SMMC-7721 cells.** (**A**) Combinational epigenetic drug treatment in MHCC97L cells with 10 µM 5-Aza-dC, 0.25 µg/mL TSA and 10µM DZNep induced the most robust DLC1 re-expression than any single or dual epigenetic drugs treatment. DZNep and 5-Aza-dC treatment were performed for 72 hours. TSA was either added to cells alone or with other drugs during the last 24 hours of the treatment. Data are represented as mean ± SEM from three independent experiments. (**B**) Addition of DZNep in SMMC-7721 cells did not further induce DLC1 re-expression when compared to 5-Aza-dC and TSA dual treatment. Data are represented as mean ± SEM from three independent experiments.

Since multiple epigenetic repressive machineries are intimately linked to establish a less permissive chromatin environment to suppress gene transcription [36,37], we sought to demonstrate the combinational effect of different epigenetic machineries in controlling DLC1 expression. MHCC97L and SMMC-7721 cells were treated with DZNep together with 5-Aza-2’deoxycytidine (5-Aza-dC) and Trichostatin A (TSA), which are well characterized DNA methylation and histone acetylation inhibitors, respectively. While treatment of DZNep, 5-Aza-dC and TSA individually elevated expression DLC1, combined treatment of three drugs synergistically restored DLC1 expression in MHCC97L cells (Figure 4A). However, the co-treatment in SMMC-7721 cells did not show further increase in DLC1 expression as compared to 5-Aza-dC alone (Figure 4B), implying that DNA methylation was a predominant epigenetic mechanism for silencing DLC1 in this cell line.

### DZNep treatment disrupts actin cytoskeleton to inhibit HCC cell migration

**Figure 5:**
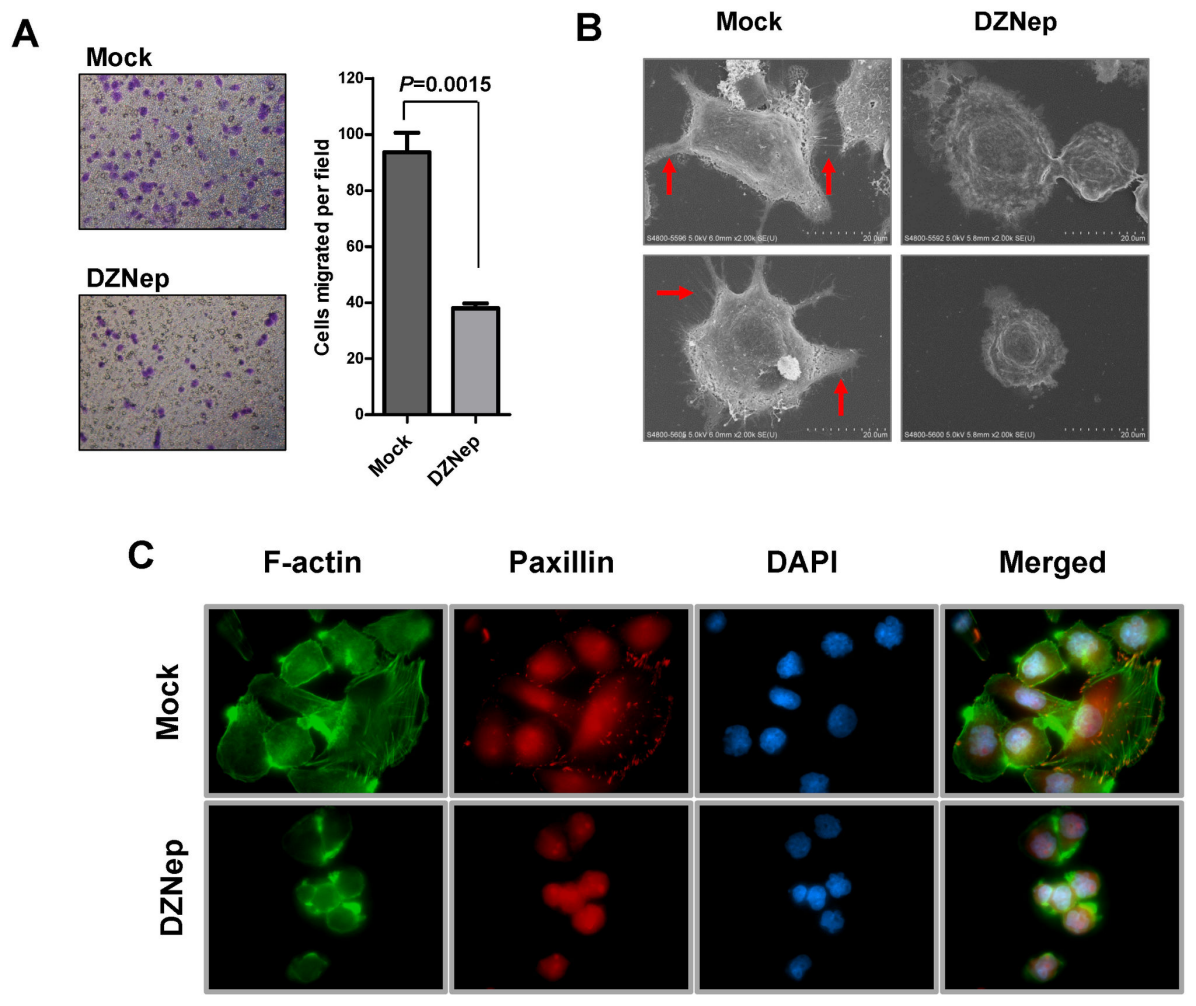
**Treatment of DZNep inhibited HCC cell migration through disruption of actin cytoskeleton.** (**A**) DZNep treatment effectively abolished MHCC97L cell migratory ability. Prior to cell migration assay, cells were treated with 1µM DZNep for 48 hours. Mock and DZNep-treated cells were then subject to cell migration assay using Transwell apparatus. Representative images of three independent experiments were shown. *P*-values obtained from *t*-test. (**B**) DZNep treatment suppressed formation of filopodia (red arrows) and lamellipodia (blue arrows) as illustrated by scanning electron microscopy. Images (2000x magnification) were captured using a Hitachi S-4800 FEG Scanning Electron Microscope. (**C**) DZNep treatment impaired actin cytoskeleton and caused cell shrinkage in MHCC97L cells. Stress fiber was stained with FITC-conjugated phalloidin and focal adhesions were stained with anti-paxillin antibody. Nuclei were counterstained with DAPI. Mock-treated cells showed organized bundles of stress fibers and well attached paxillin. DZNep-treated cells shrank and lost proper actin cytoskeleton network. Images (100x magnification) were captured by a Leica Q550CW fluorescence microscope (Leica, Wetzler, Germany).

DLC1/Rho/ROCK pathway is crucial for regulating proper cytoskeleton remodeling and cell motility. Our proceeding findings suggest that EZH2 is involved in suppressing DLC1, we therefore further tested whether DZNep treatment could suppress *in vitro* HCC migration. DZNep treatment at 1µM for 48 hours, which showed minimal cytotoxic effect (Supplementary Figure S2), effectively inhibited cell migration in MHCC97L cells as demonstrated by Transwell migration assay (Figure 5A). DZNep treated cells also showed abolishment of proper actin cytoskeleton network when examined under scanning electron microscope (Figure 5B) and immunofluorescence staining (Figure 5C). These cell morphological alterations induced by treatment with DZNep highly resembled those of DLC1-induced cell cytoskeletal collapse and cell shrinkage [21], implying that DZNep may inhibit cell migration via interfering DLC1/ROCK pathway and caused substantial actin cytoskeleton disorganization.

## Discussion

One important pro-metastatic role of EZH2 in cancer is via epigenetic silencing of tumor and metastasis suppressor genes [8–12]. H3K27me3 is the best characterized EZH2-mediated histone modification in the mammalian epigenetic system. In the present study, we provide evidence that EZH2-mediated H3K27me3 is involved in the epigenetic repression of DLC1 during HCC development. Interestingly, from several published genome-wide data of Polycomb group (PcG) target gene mapping in human embryonic stem (hES) cells, we also noticed that DLC1 is a candidate PcG-regulated gene in early developmental stage (Supplementary Figure S3). In hES cells, the DLC1 promoter is occupied by SUZ12, which is associated with EZH2 to form the PRC2 in mediating H3K27me3 and transcription repression [38]. In addition, the DLC1 promoter is also marked with H3K27me3 [39] and H3K4me3 [40], which constitute the bivalent chromatin signature of PcG targets [41]. This bivalent chromatin signature, involving the H3K27me3-silencing and H3K4me3-activating histone modifications, allows genes of developmental importance and genes required for stemness maintenance to be poised in a ready-to-transcribe state until differentiation [41,42]. DLC1 is essential for embryonic development [43] and possibly has a role in lineage specification [44]. DLC1 knockout mice is embryonic lethal [43]. However, DLC1 is ubiquitously transcribed in adult tissues [14], implying that PcG silencing effect has been relieved in differentiated somatic cells. Upon oncogenesis, it is possible that DLC1 regains its embryonic PcG marking as a result of EZH2 up-regulation. At the molecular level, we demonstrated that DLC1 was co-regulated by EZH2-mediated H3K27me3, DNA methylation and histone deacetylation in HCC cells. However, the co-regulation between the three epigenetic mechanisms on DLC1 was only observed when its promoter was partially methylated (such as in MHCC97L cells). Once DLC1 promoter was densely methylated (such as in SMMC-7721 cells), H3K27me3 was absent and DNA methylation was the key repressive mechanism with modest participation of histone deacetylation. This observation may reflect the crosstalk of EZH2 silencing and DNA methylation in an earlier stage of epigenetic repression of tumor suppressor genes before the eventual replacement of H3K27me3 silencing by the stable DNA methylation machinery [45,46]. Indeed, our findings that DLC1 is a PcG-regulated target and its subsequent epigenetic silencing during malignancy progression are also in line with the recently proposed model on PcG marking of genes in ES cells are predisposed to *de novo* cancer specific DNA methylation [45,46].

Pharmacologic targeting of deregulated epigenetic proteins emerges as an attractive approach in cancer therapy [47,48]. DZNep has been shown to eradicate tumor-initiating HCC cells in nude mice implanted with HCC [49], induce apoptosis in breast cancer cells [29] and target human acute myeloid leukemia in mice model when used in combination with panobinostat [50]. However, the metastasis suppressive effect of DZNep has never been evaluated. Our findings indicate that DZNep inhibit EZH2 expression and may further function as a potent metastasis inhibitor through disrupting actin cytoskeleton organization. A detailed pharmacological characterization of DZNep to suppress HCC tumor growth and progression in vivo is still warranted to investigate the therapeutic potential of DZNep as cancer treatment regimen.

We have previously showed that EHZ2 promotes HCC metastasis in part through epigenetic repression of multiple Rho/ROCK signaling-targeting miRNAs [13]. For example, miR-139 that targets ROCK2 [22] was frequently down-regulated in human HCC by EZH2-mediated H3K27me3 [13]. In this study, we additionally demonstrated that DLC1, the key up-stream regulator of Rho/ROCK pathway, is also silenced by EZH2. Altogether, our findings unravel a tight and multilayered regulation of DLC1/Rho/ROCK signaling by EZH2, which may underpin a critical epigenetic driven event in promoting cancer metastasis.

## Supporting Information

Figure S1 Transient overexpression of GFP-EZH2, as confirmed by immunoblotting (left panal) and qPCR (right panal), augmented the EZH2 and H3K27me3 levels in Huh-7 cells. Anti-EZH2 antibody (#3147, Cell Signaling Technology, Danvers, MA, USA) detected both the endogenous and GFP-EZH2 fusion protein in the immunoblot. Protein and RNA were harvested six-day post transfection.
(TIF)

Figure S2 MHCC97L cells treated with 1µM DZNep for 48 hours were examined for their viability by trypan blue staining prior to cell migration assay. Mock and DZNep treated cells were similarly viable.
(TIF)

Figure S3 Publicly available ChIP-sequencing and ChIP-on-chip database was analyzed to provide clues of DLC1 chromatin status in human embryonic stem cell. DLC1 locus was found to be marked by H3K27me3 (Zhao et al., 2007) and H3K4me3 (Pan et al., 2007) in independent studies. Furthermore, DLC1 promoter was also found to be bound by SUZ12 (Lee et al., 2006), which is a core component of the Polycomb Repressive Complex 2.
(TIF)
