# Correction: EZH2-Mediated H3K27me3 Is Involved in Epigenetic Repression of Deleted in Liver Cancer 1 in Human Cancers

**DOI:** 10.1371/annotation/9085e9bd-25b1-4266-b6db-34e56d80ce3a

**Published:** 2014-01-24

**Authors:** Sandy Leung-Kuen Au, Carmen Chak-Lui Wong, Joyce Man-Fong Lee, Chun-Ming Wong, Irene Oi-Lin Ng

An error occurred which caused only one of the two designated corresponding authors to appear. Dr. Chun-Ming Wong is also a corresponding author and can be contacted at jackwong@pathology.hku.hk.

