# Supplementary figures and images for: EZH2-Mediated H3K27me3 Is Involved in Epigenetic Repression of Deleted in Liver Cancer 1 in Human Cancers
Source: PLoS One. 2013 Jun 27;8(6):e68226. doi: 10.1371/journal.pone.0068226 (PMC3694912; doi:10.1371/journal.pone.0068226)

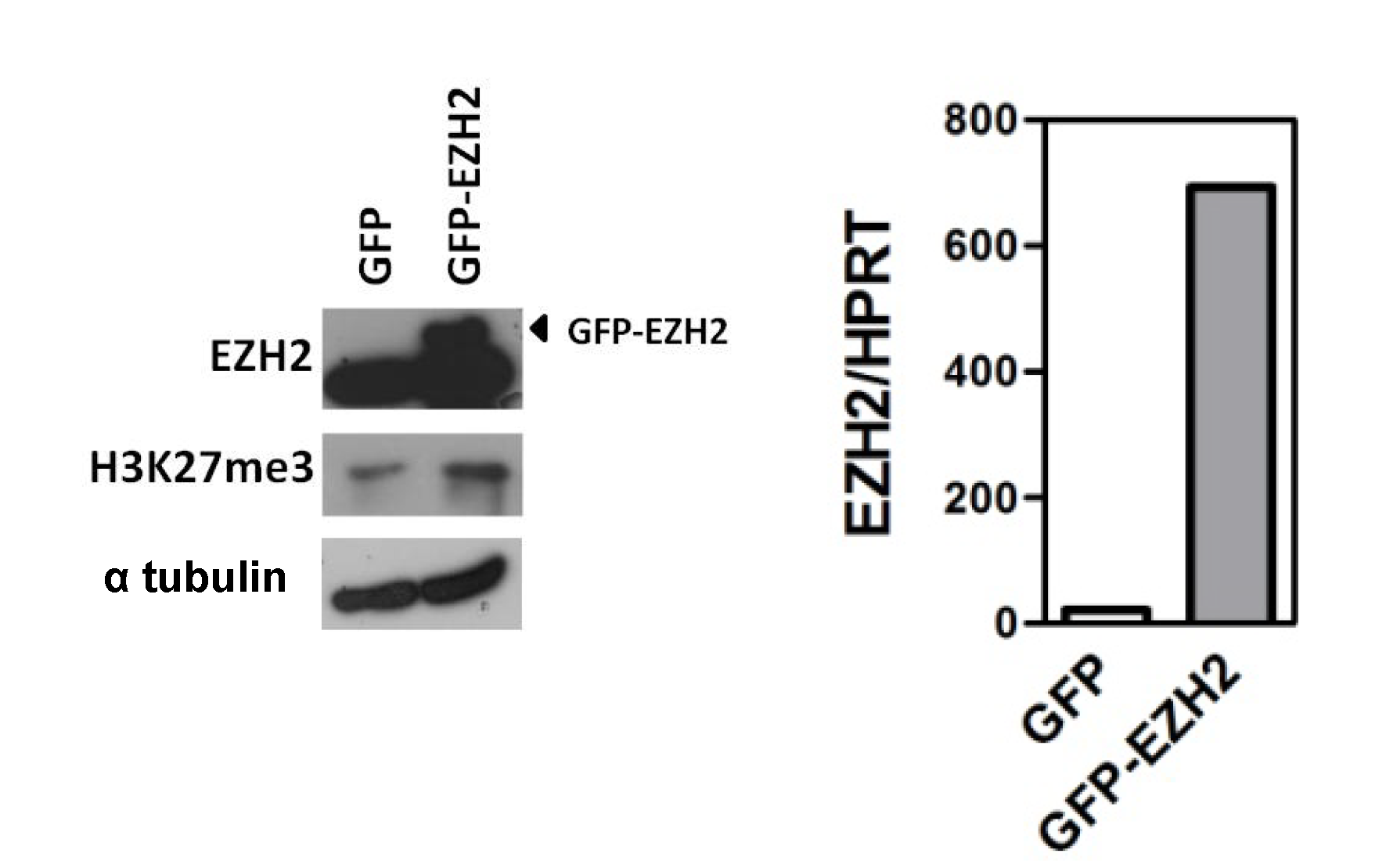

Supplement: Figure S1 — Transient overexpression of GFP-EZH2, as confirmed by immunoblotting (left panal) and qPCR (right panal), augmented the EZH2 and H3K27me3 levels in Huh-7 cells. Anti-EZH2 antibody (#3147, Cell Signaling Technology, Danvers, MA, USA) detected both the endogenous and GFP-EZH2 fusion protein in the immunoblot. Protein and RNA were harvested six-day post transfection. (TIF) [file pone.0068226.s001.tif]

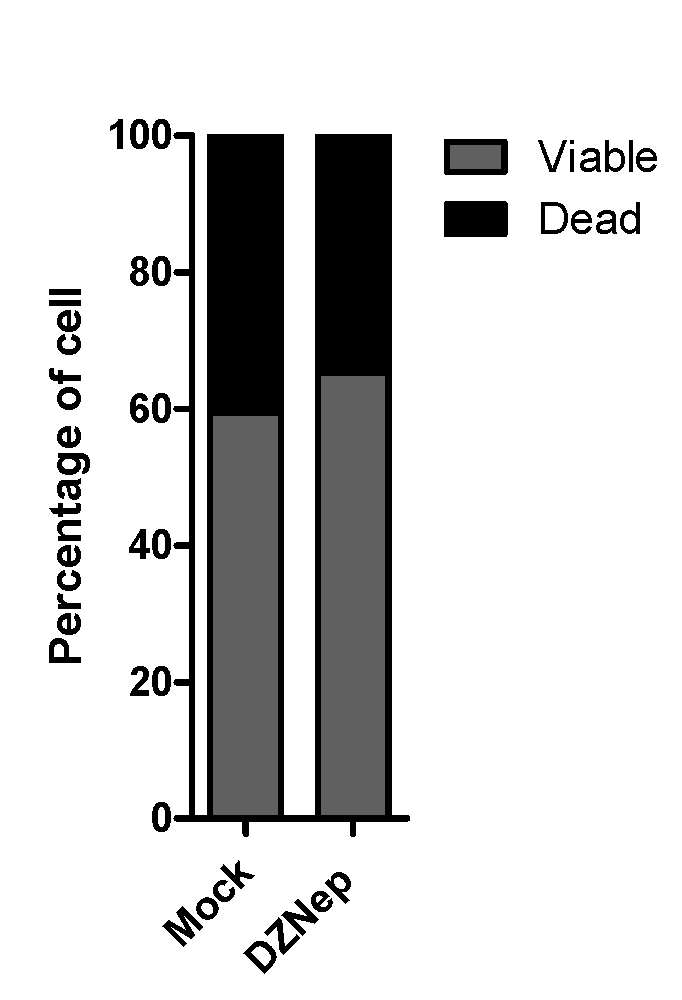

Supplement: Figure S2 — MHCC97L cells treated with 1µM DZNep for 48 hours were examined for their viability by trypan blue staining prior to cell migration assay. Mock and DZNep treated cells were similarly viable. (TIF) [file pone.0068226.s002.tif]

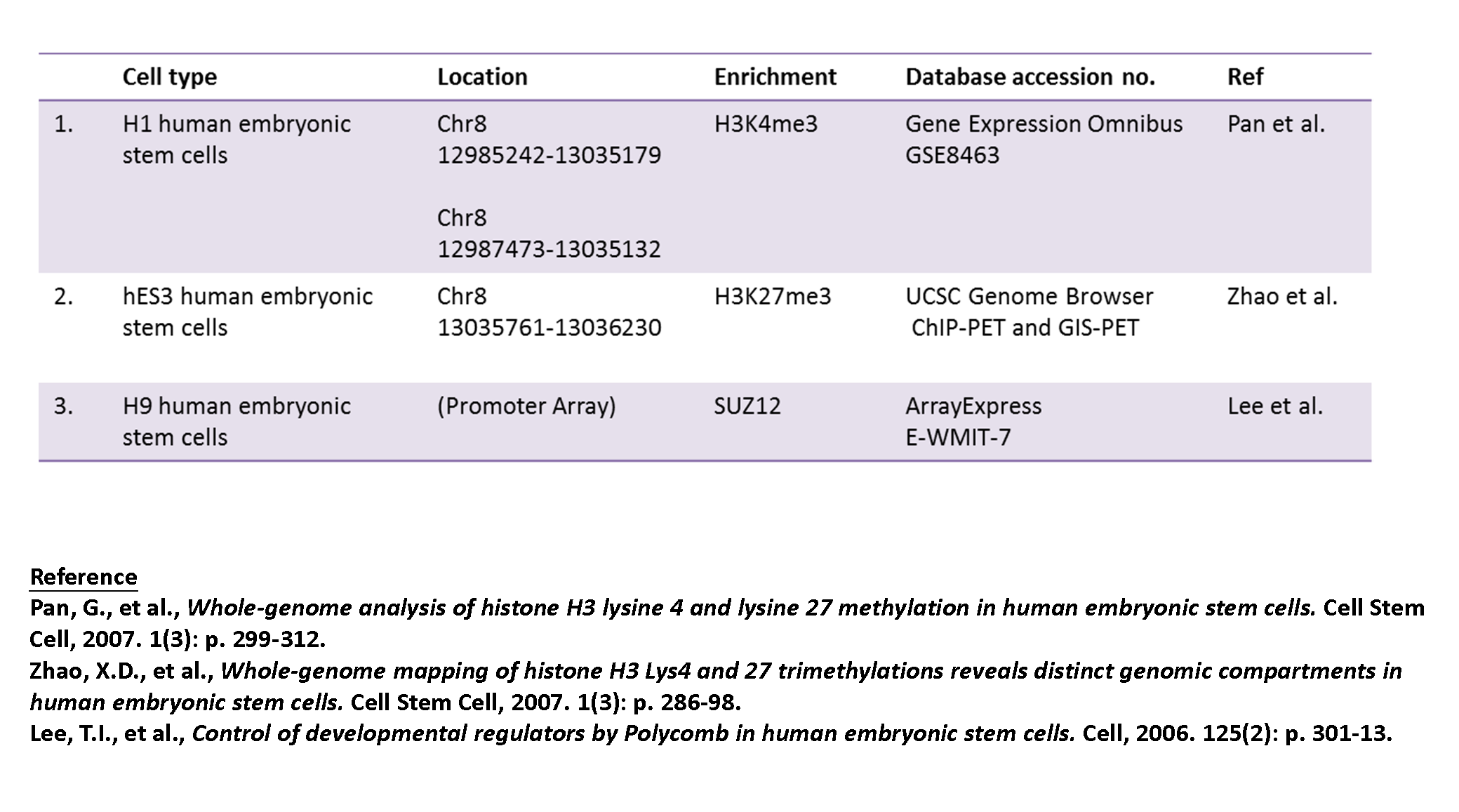

Supplement: Figure S3 — Publicly available ChIP-sequencing and ChIP-on-chip database was analyzed to provide clues of DLC1 chromatin status in human embryonic stem cell. DLC1 locus was found to be marked by H3K27me3 (Zhao et al., 2007) and H3K4me3 (Pan et al., 2007) in independent studies. Furthermore, DLC1 promoter was also found to be bound by SUZ12 (Lee et al., 2006), which is a core component of the Polycomb Repressive Complex 2. (TIF) [file pone.0068226.s003.tif]
